# Text Message Reminders to Improve Immunization Appointment Attendance in Alberta, Canada: The Childhood Immunization Reminder Project Pilot Study

**DOI:** 10.2196/37579

**Published:** 2022-11-08

**Authors:** Shannon E MacDonald, Emmanuel Marfo, Hannah Sell, Ali Assi, Andrew Frank-Wilson, Katherine Atkinson, James D Kellner, Deborah McNeil, Kristin Klein, Lawrence W Svenson

**Affiliations:** 1 Faculty of Nursing University of Alberta Edmonton, AB Canada; 2 School of Public Health University of Alberta Edmonton, AB Canada; 3 South Zone Data & Analytics Alberta Health Services Lethbridge, AB Canada; 4 Faculty of Health Sciences University of Lethbridge Lethbridge, AB Canada; 5 Department of Global Public Health Karolinska Institutet Stockholm Sweden; 6 Clinical Epidemiology Program Ottawa Hospital Research Institute Ottawa, ON Canada; 7 Department of Pediatrics University of Calgary Calgary, AB Canada; 8 Alberta Health Services Calgary, AB Canada; 9 Strategic Clinical Networks Alberta Health Services Calgary, AB Canada; 10 Communicable Disease Control Provincial Population & Public Health Alberta Health Services Edmonton, AB Canada; 11 Division of Preventive Medicine Department of Medicine University of Alberta Edmonton, AB Canada; 12 Analytics and Performance Reporting Alberta Health Edmonton, AB Canada; 13 Department of Community Health Sciences University of Calgary Calgary, AB Canada

**Keywords:** text message, SMS, immunization reminder, reminder-recall, routine immunization, childhood, immunization, reminder, children, language barrier, Canada, vaccine, vaccination, coverage

## Abstract

**Background:**

Vaccine coverage for 18-month-old children in Canada is often below the recommended level, which may be partially because of parental forgetfulness. SMS text message reminders have been shown to potentially improve childhood immunization uptake but have not been widely used in Alberta, Canada. In addition, it has been noted that language barriers may impede immunization service delivery but continue to remain unaddressed in many existing reminder and recall systems.

**Objective:**

This study aimed to assess the effectiveness and acceptability of using SMS text messages containing a link to web-based immunization information in different languages to remind parents of their child’s 18-month immunization appointment.

**Methods:**

The Childhood Immunization Reminder Project was a pilot intervention at 2 public health centers, one each in Lethbridge and Edmonton, Alberta, Canada. Two SMS text message reminders were sent to parents: a booking reminder 3 months before their child turned 18 months old and an appointment reminder 3 days before their scheduled appointment. Booking reminders included a link to the study website hosting immunization information in 9 languages. To evaluate intervention effectiveness, we compared the absolute attendance no-show rates before the intervention and after the intervention. The acceptability of the intervention was evaluated through web-based surveys completed by parents and public health center staff. Google Analytics was used to determine how often web-based immunization information was accessed, from where, and in which languages.

**Results:**

Following the intervention, the health center in Edmonton had a reduction of 6.4% (95% CI 3%-9.8%) in appointment no-shows, with no change at the Lethbridge Health Center (0.8%, 95% CI −1.4% to 3%). The acceptability surveys were completed by 222 parents (response rate: 23.9%) and 22 staff members. Almost all (>95%) respondents indicated that the reminders were helpful and provided useful suggestions for improvement. All surveyed parents (222/222, 100%) found it helpful to read web-based immunization information in their language of choice. Google Analytics data showed that immunization information was most often read in English (118/207, 57%), Punjabi (52/207, 25.1%), Arabic (13/207, 6.3%), Spanish (12/207, 5.8%), Italian (4/207, 1.9%), Chinese (4/207, 1.9%), French (2/207, 0.9%), Tagalog (1/207, 0.5%), and Vietnamese (1/207, 0.5%).

**Conclusions:**

The study’s findings support the use of SMS text message reminders as a convenient and acceptable method to minimize parental forgetfulness and potentially reduce appointment no-shows. The diverse languages accessed in web-based immunization information suggest the need to provide appropriate translated immunization information. Further research is needed to evaluate the impact of SMS text message reminders on childhood immunization coverage in different settings.

## Introduction

### Background

Immunization is a safe and cost-effective intervention that substantially reduces childhood morbidity and mortality [1]. Routine childhood immunization is publicly funded across Canada. However, childhood vaccine coverage remains suboptimal [2]. Evidence from Canada and globally has shown a link between suboptimal vaccine coverage and vaccine-preventable disease outbreaks [3,4].

It is a well-recognized phenomenon that the uptake of infant vaccines exhibits a precipitous drop at the 18-month vaccine doses [5-7]. The vaccines administered at 18 months of age in Canada include diphtheria-tetanus-acellular pertussis-polio-*Haemophilus influenzae* type b (DTaP-IPV-Hib) in all provinces or territories, measles-mumps-rubella-varicella (MMRV) in 7 provinces or territories, and pneumococcal conjugate 13-valent (Pneu C13) and hepatitis B in one province or territory each [8]. The drop in coverage at 18 months is exemplified in Alberta, where 2021 coverage levels for the third dose of DTaP-IPV-Hib, typically given at 6 months, was 89%, but only 75% for the fourth dose, given at 18 months [9].

The literature has shown that parental forgetfulness of immunization appointments is a key barrier to 18-month vaccine uptake [10,11]. Factors contributing to this forgetfulness in Canadian parents include the following: (1) the perception that infant vaccines are completed by 12 months of age, (2) the end of paid parental leave and return to work, (3) a 6-month gap between appointments, and (4) the inability of some booking systems to schedule an appointment 6 months in advance [12,13]. People with low socioeconomic status are more likely to have low vaccine coverage because of challenges in caring for multiple children, multiple household moves, inadequate income, and language barriers [14]. Suboptimal immunization coverage among certain populations is problematic and creates work for health care providers in catching up with missed doses. Hence, there is a need for a robust immunization appointment reminder system to help alleviate some of these challenges.

Previous research has shown that SMS text message reminders improve childhood immunization uptake [3,15], particularly when educational information is included [16,17]. Furthermore, providing educational information in different languages can promote engagement with immunization information and help parents of different ethnic backgrounds understand immunization benefits [18]. SMS text messaging has also been shown to be relatively low cost, technologically easy, widely available, and applicable to various health problems [19,20].

### Objectives

Given that the effectiveness of public health interventions is context specific [21], assessing whether a new SMS text message intervention would have the intended impact (ie, fewer missed appointments) and would be acceptable to stakeholders is crucial. There was interest among public health stakeholders in Alberta in testing an SMS text messaging reminder system for preschool immunization. Thus, this study aimed to assess the effectiveness and acceptability of using SMS text messages containing a link to web-based immunization information in different languages to remind parents of their child’s 18-month immunization appointment.

## Methods

### Setting

Alberta is a western Canadian province with approximately 4.4 million residents. The province is divided into 5 zones for the administration of health services by Alberta Health Services (AHS). Routine preschool immunization is delivered exclusively by nurses at public health centers (PHCs).

### Intervention

The Childhood Immunization Reminder Project (ChIRP) was a pilot intervention aimed at improving attendance at 18-month immunization appointments by sending SMS text message reminders to parents. ChIRP was implemented at two PHCs: (1) Mill Woods PHC in the Edmonton health zone, which serves an ethnically diverse population in a high-density urban city with a total population of 1.1 million [22], and (2) Lethbridge PHC in the South Health Zone, which serves a more ethnically homogenous population in a small city within a rural area with a population of 102,000 [22].

Using an automated system (iceAlert), 2 SMS text message reminders were sent to each parent participating in the study. Messages were sent to the primary phone number on file provided by the parents during registration. Most were cell phone numbers, but voice message reminders were sent instead of SMS text messages if the phone number provided was a landline number. The first message was sent 3 months before the child turned 18 months (ie, when the child was 15 months old) to remind parents to book the 18-month appointment or to reschedule an existing appointment if needed. The reminders were sent monthly between May 2019 and May 2020 to all parents of children who turned 15 months old in that month and had a postal code within the service area of Mill Woods or Lethbridge PHCs. This reminder included a link to the study website hosting immunization information in 9 languages (English, French, Arabic, traditional Chinese, Italian, Punjabi, Spanish, Tagalog, and Vietnamese).

**Figure 1:**
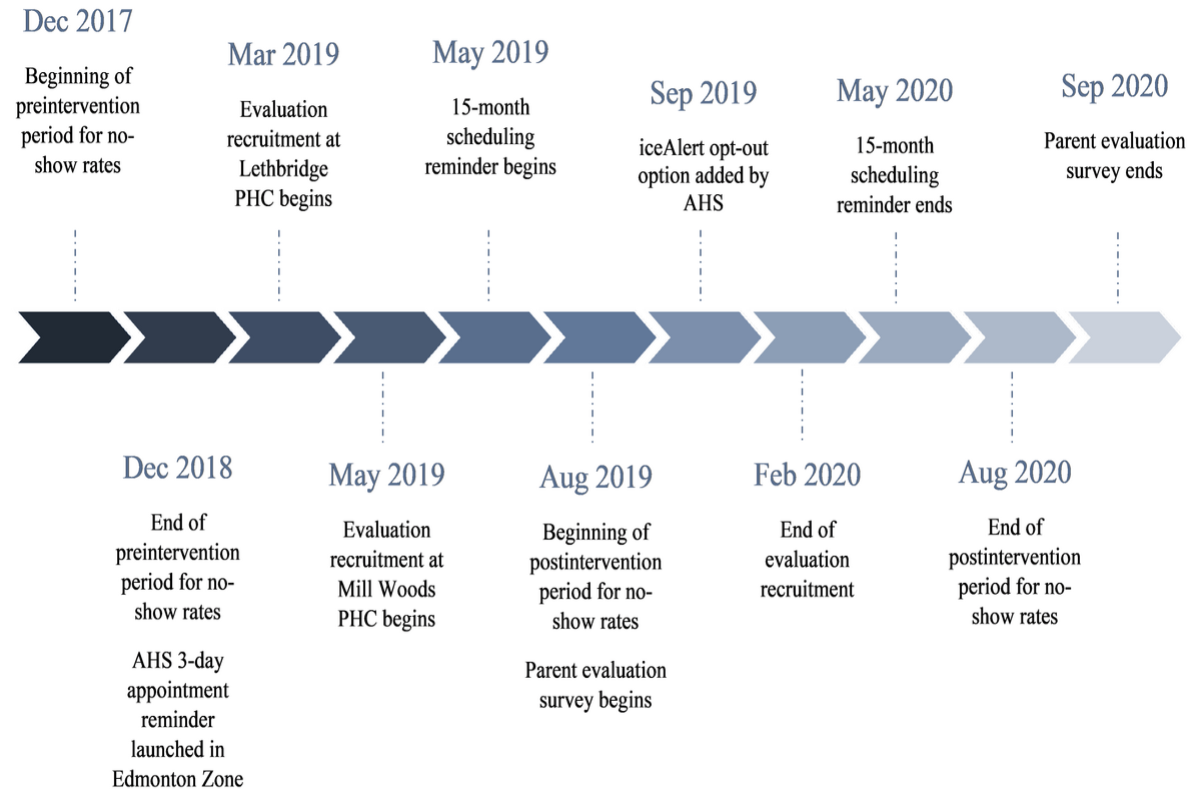
Timeline of study intervention and evaluation periods. AHS: Alberta Health Services; PHC: public health center.

The second message was a reminder of the date and time of the booked 18-month appointment, which was sent 3 days before the scheduled visit. This reminder was initiated by AHS in December 2018, shortly before the start of ChIRP, and was, therefore, incorporated into the evaluation. This was an opt-out system (ie, parents received the message unless they asked not to). Figure 1 shows the timeline of the intervention and evaluation periods.

### Evaluation

#### Effectiveness

To evaluate the effectiveness of the intervention, we compared absolute no-show rates before (December 2017 to December 2018) and after (August 2019 to August 2020) the intervention in both PHCs using routinely collected administrative data from AHS. No-shows were defined as children who had missed their scheduled appointments, including those who rescheduled missed appointments. We identified all children with 18-month immunization appointments at the PHCs by using provincial patient identification numbers. Children were excluded from the analysis if their appointment was canceled or outside the preintervention or postintervention periods, they had not yet had their appointment, or were aged >24 months. No-show rates were determined by dividing the total number of no-shows by the total number of 18-month immunization appointments at each PHC. The no-show rates at the intervention sites were compared with PHCs with similar client demographics in the same health zone (ie, Northeast Edmonton PHC for Mill Woods and Medicine Hat PHC for Lethbridge) using a 2-sample proportion test. The analysis was performed using R (version 3.6.3; R Foundation for Statistical Computing) [23] and Stata (version 15.1; StataCorp).

#### Acceptability

Parents and PHC staff evaluated the acceptability of the reminder intervention. The PHC staff recruited parents for the web-based survey evaluation during their child’s 12-month immunization appointment, starting in March (Lethbridge) or May 2019 (Edmonton) and continuing until February 2020. Interested parents received an information sheet and consent form (available in 9 languages) that collected their name, child’s name and date of birth, and mobile phone number. There were no eligibility restrictions in terms of age, gender, or other sociodemographic characteristics other than needing a mobile phone to receive the evaluation survey link. In consultation with PHC managers, a parallel survey was sent to all staff who worked at either participating PHC during the intervention period, including nurses and clerical staff. Informed consent was obtained from all the participants.

Participating parents were sent a text message containing a link to a web-based survey when their child was 19 months old, after the intended 18-month immunization appointment. Parent surveys were completed between September 2019 and October 2020. The PHC staff were sent the staff survey link via email at the end of the intervention period in December 2020 or January 2021. Survey data were collected and managed using REDCap (Research Electronic Data Capture; Vanderbilt University) tools hosted and supported by the Women and Children’s Health Research Institute at the University of Alberta [24].

Sociodemographic information collected from parents included residential location, whether they were born in Canada, language or languages read, education level, and annual household income. The PHC staff provided the PHC’s location and their job position. Parents were asked about the helpfulness of the reminders, when the reminders should be sent, what actions they took because of the reminders, and whether their child had attended the 18-month immunization appointment. The PHC staff evaluated the helpfulness and impact of the reminders, when and how many reminders should be sent, and which other routine vaccine programs (2-month, 4-month, 6-month, 12-month, preschool, and school-based) should be considered for reminders.

Both parents and PHC staff evaluated the helpfulness and impact of the web-based immunization information included in the reminder. Google Analytics [25] was used to determine how often the information was accessed, from where, and in which languages. The survey data were analyzed using SPSS (version 26; IBM Corp) [26]. Descriptive statistics (ie, frequencies or percentages) were calculated for the survey responses.

### Ethics Approval

This study was approved by the Health Research Ethics Board of the University of Alberta (study ID: Pro00085642).

## Results

### Overview

Throughout the intervention period (May 2019 to May 2020), a total of 3307 booking reminders were successfully sent, including 2885 SMS text messages and 422 voice notifications. A small number of reminders (n=133) were not delivered. Data on the number of 3-day appointment reminders sent were not available because these reminders were sent zone-wide by AHS.

### Effectiveness

After removing those who had not yet had an appointment or had incomplete data, the Mill Woods PHC had 638 appointments for 18-month immunizations during the preintervention period. Of the 638 appointments, 116 (18.2%) were either no-show or initially no-show and then rebooked. During the postintervention period, there were 1508 appointments for 18-month immunizations, with 178 (11.8%) no-shows. Data from the Northeast Edmonton PHC are shown for comparison (Table 1). Between the preintervention and postintervention periods, Mill Woods experienced a 6.4% (95% CI 3.0%-9.8%) decline in absolute no-show rates, significantly more than the control site in the Northeast Edmonton PHC (Table 1).

At Lethbridge PHC, there were 1657 appointments for 18-month immunization during the preintervention period, 186 (11.22%) of which were no-shows. During the postintervention period, there were 1653 appointments and 198 (11.97%) no-shows. Data from the Medicine Hat PHC are shown for comparison. There were no significant differences between preintervention and postintervention no-show rates in the other intervention (Lethbridge) and control site (Medicine Hat).

**Table 1:** Absolute no-show^a^ rates and change in rates before the intervention (December 2017 to December 2018) and after the intervention (August 2019 to August 2020).

Health zone				Preintervention rates					Postintervention rates					Change		
				Rate, n (%)		95% CI		Rate, n (%)			95% CI		Rate, %^b^			95% CI
**Edmonton zone**																
	**Mill Woods (intervention site)**															
		Attended	522 (81.8)		78.8 to 84.8		1330 (88.2)			86.6 to 89.8		6.4			3.0 to 9.8	
		No-show	116 (18.2)		15.2 to 21.2		178 (11.8)			10.2 to 13.4		−6.4			−9.8 to −3.0	
	**Northeast Edmonton (control site)**															
		Attended	388 (85.7)		82.5 to 88.9		1105 (81.9)			79.8 to 84.0		−3.8			−7.6 to 0.0	
		No-show	65 (14.3)		11.1 to 17.5		244 (18.1)			16.0 to 20.2		3.8			0.0 to 7.6	
**South zone**																
	**Lethbridge (intervention site)**															
		Attended	1471 (88.8)		87.3 to 90.3		1455 (88)			86.4 to 89.6		−0.8			−3.0 to 1.4	
		No-show	186 (11.2)		9.7 to 12.7		198 (12)			6.0 to 13.6		0.8			−1.4 to 3.0	
	**Medicine Hat (control site)**															
		Attended	904 (89)		87.1 to 90.9		824 (88.1)			86.0 to 90.2		−0.9			−3.7 to 1.9	
		No-show	112 (11)		9.1 to 12.9		111 (11.9)			5.4 to 14.0		0.9			−1.9 to 3.7	

^a^No-show was defined as when a client failed to turn up for their scheduled appointment, including those who initially did not turn up for their scheduled appointment and later rebooked it.

^b^Change in rates calculated as the difference between postintervention and preintervention rates.

### Acceptability

A total of 929 parents consented to participate in the evaluation survey (Mill Woods, n=484; Lethbridge, n=445), whereas 107 declined to participate (Mill Woods, n=24; Lethbridge, n=83). Of those who consented, 222 completed the parent survey (Mill Woods, n=105; Lethbridge, n=117) and 10 declined the survey after receiving it (Mill Woods, n=7; Lethbridge, n=3), with a response rate of 23.9% (222/929). Of those who responded, 93.7% (208/222) reported attending the 18-month visit, whereas 6.3% (14/222) reported missing it. A total of 22 PHC staff members completed the staff survey (Mill Woods, n=12; Lethbridge, n=10). The number who received the invitation was not available, as the PHC managers were responsible for forwarding the invitation to all staff who worked during the intervention period.

### Sociodemographic Characteristics

As seen in Table 2, a total of 51.8% (115/222) of the parent sample was located in or near Lethbridge and 46.8% (104/222) was located in or near Mill Woods. Most were born in Canada (203/222, 91.4%), were most comfortable reading English (207/222, 93.2%), had a university degree (104/222, 46.8%) or a college certificate or diploma (87/222, 39.2%), and had an annual household income of greater than CAD $90,000 (US $65,894.85; 77/222, 34.7%). Slightly over half of the PHC staff sample was employed at Mill Woods (12/22, 55%), with the remainder employed at Lethbridge (10/22, 46%). Most of the surveyed staff members were nurses (19/22, 86%).

**Table 2:** Sociodemographic characteristics of parents (n=222) and public health center staff (n=22).

Variable				Respondents, n (%)
**Parents**				
	**Location**			
		In or near Lethbridge	115 (51.8)	
		In or near Mill Woods (Edmonton)	104 (46.8)	
		Not specified	3 (1.4)	
	**Arrived in Canada in the last 5 years**			
		Yes	16 (7.2)	
		No	197 (88.7)	
		No response	9 (4.1)	
	**Language most comfortable reading**			
		English	207 (93.2)	
		Spanish	4 (1.8)	
		Punjabi	2 (0.9)	
		Chinese	1 (0.5)	
		Others^a^	7 (3.1)	
		No response	1 (0.5)	
	**Highest level of education completed**			
		University degree	104 (46.8)	
		College or other post–high-school academic certificate or diploma	87 (39.2)	
		High school	21 (9.5)	
		Lower than high school	1 (0.5)	
		Prefer not to answer or no response	9 (4)	
	**Annual household income (CAD $)^b^**			
		<30,000	22 (9.9)	
		30,000-59,999	36 (16.2)	
		60,000-89,999	42 (18.9)	
		>90,000	77 (34.7)	
		Prefer not to answer	33 (14.9)	
		Do not know	6 (2.7)	
		No response	6 (2.7)	
**Public health center staff**				
	**Health center location**			
		Mill Woods (Edmonton zone)	12 (55)	
		Lethbridge (south zone)	10 (46)	
	**Job position**			
		Nurse	19 (86)	
		Manager	2 (9)	
		Administrative support	1 (5)	

^a^Other languages included Arabic, Dinka, Somali, Swedish, Ukrainian, Urdu, Yoruba, and not specified (all n=1).

^b^At the time of study, CAD $1 was approximately equal to US $0.73.

### Fifteen-Month Booking Reminder

In total, 51.4% (114/222) of the parents surveyed reported receiving the 15-month booking reminder (Table 3). Of these 114 parents, 96.5% (n=110) reported that it was helpful. Of the 110 parents who found the reminder helpful, 30.9% (n=34) booked or rescheduled their child’s 18-month immunization appointment after receiving the reminder. Of all surveyed parents (N=222), most reported that a reminder to book the 18-month immunization appointment should be sent when children were 17 months old (n=114, 51.4%) rather than when they were 15 months old (when it was delivered for the study).

As shown in Table 3, most PHC staff members reported that the 15-month reminder was helpful (21/22, 96%). A total of 81% (18/22) of the staff reported that more clients came to their scheduled appointments than usual during the 1-year intervention period. Staff from the 2 PHCs had different preferences for when to send the booking reminder; the Mill Woods staff preferred the reminder be sent at 15 months (41.7%) while Lethbridge staff preferred 16 months (6/10, 60%). Most staff members at both PHCs reported that 2 immunization booking reminders should be sent to clients (12/22, 55%).

**Figure 2:**
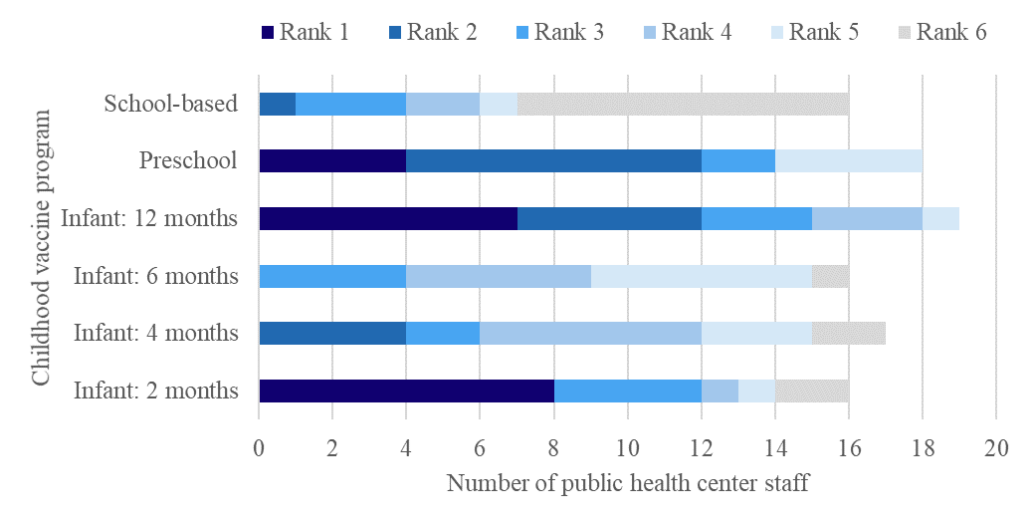
Public health center staff (n=19) rankings of childhood vaccine programs for priority of scheduling or booking reminders (of those who answered yes to whether reminders will be helpful for other routine vaccine programs).

As shown in Figure 2, a total of 19 PHC staff members (Mill Woods, n=11; Lethbridge, n=8) ranked childhood vaccine programs for booking reminders according to their priority. One participant from Mill Woods and 2 participants from Lethbridge did not provide a ranking. Of the 19 participants who provided rankings, some provided <6 rankings, so the total for each ranking may not add up to 19. The 2-month (8/19, 42%) and 12-month (7/19, 37%) vaccine programs were most commonly ranked first for scheduling or booking reminders, followed by the preschool program (4/19, 21%). Almost half of the PHC staff ranked the school-based vaccine program as the lowest priority (9/19, 47%).

**Table 3:** Evaluation of the 15-month booking reminder by parents who reported receiving the 15-month reminder (n=114) and public health center staff who completed the survey (n=22).

Variable			Response, n (%)		
			Mill Woods	Lethbridge	Total
**Parents**					
	**15-month reminder was helpful (n=114)**				
		Yes	57 (96.6)	53 (96.4)	110 (96.5)
		No	1 (1.7)	1 (1.8)	2 (1.8)
		I do not know	1 (1.7)	1 (1.8)	2 (1.7)
	**Impact of the 15-month reminder (n=110)^a^**				
		Booked appointment	14 (24.6)	14 (26.4)	28 (25.5)
		Changed appointment	4 (7)	2 (3.8)	6 (5.5)
		Did nothing	37 (64.9)	34 (64.2)	71 (64.5)
		Forgot to book or reschedule	1 (1.8)	2 (3.8)	3 (2.7)
		Other	1 (1.8)	1 (1.9)	2 (1.8)
	**Why the 5-month reminder was helpful (n=110)^a,b^**				
		Reminded of the appointment	55 (96.5)	53 (100)	108 (98.2)
		Specified the name of the child	15 (26.3)	16 (30.2)	31 (28.2)
		Included phone number to call for booking	18 (31.6)	24 (45.3)	42 (38.2)
		Included vaccine information link	5 (8.8)	14 (26.4)	19 (17.3)
	**Best time to send the reminder (n=222)^c^**				
		When child is 15 months	32 (30.5)	31 (26.5)	63 (28.4)
		When child is 16 months	25 (23.8)	29 (24.8)	54 (24.4)
		When child is 17 months	48 (45.7)	66 (56.4)	114 (51.4)
		Other	10 (9.5)	5 (4.3)	15 (6.8)
**Public health center staff**					
	**15-month reminder was helpful (n=22)**				
		Yes	12 (100)	9 (90)	21 (95.5)
		No	0 (0)	1 (10)	1 (4.5)
		Not sure or do not know	0 (0)	0 (0)	0 (0)
	**Impact of the 15-month reminder (n=21)^b,d^**				
		More clients came to their scheduled immunization than usual	10 (83.3)	8 (88.9)	18 (85.7)
		More clients canceled or rescheduled than usual	1 (8.3)	2 (22.2)	3 (14.3)
		Other	2 (16.7)	1 (11.1)	3 (14.3)
		No change	0 (0)	0 (0)	0 (0)
	**Best time to send the reminder (n=22)**				
		When child is 15 months	5 (41.7)	2 (20)	7 (31.8)
		When child is 16 months	4 (33.3)	6 (60)	10 (45.5)
		When child is 17 months	3 (25)	0 (0)	3 (13.7)
		No need to send a booking or rescheduling reminder	0 (0)	1 (10)	1 (4.5)
		Other	0 (0)	1 (10)	1 (4.5)
	**How many reminders should be sent (n=22)**				
		1	4 (33.3)	3 (30)	7 (31.8)
		2	6 (50)	6 (60)	12 (54.5)
		3	2 (16.7)	1 (10)	3 (13.7)

^a^For parents who responded that the 15-month reminder was helpful (Mill Woods: n=57; Lethbridge: n=53).

^b^Respondents could select more than one option.

^c^Answered by all parents, not just those who received the 15-month reminder.

^d^For staff who responded that the 15-month reminder was helpful.

### Three-Day Appointment Reminder

In total, 79.3% (176/222) of the surveyed parents reported receiving the 3-day appointment reminder (Table 4), and 6.8% (12/176) of the parents reported changing their appointments. Slightly over half (113/222, 50.9%) of the surveyed parents indicated that the best time to send reminders was 3 days before the appointment. The second preferred time to send reminders was a week before the appointment (85/222, 38.3%). Most parents (208/222, 93.6%) reported attending the 18-month immunization appointment.

Among the PHC staff, most (21/22, 96%) reported that the 3-day reminder was helpful (Table 4). Over half of the staff (12/22, 55%) indicated that the best time to send this reminder was 3 days before the visit, and 59% (13/22) of the staff indicated that only 1 reminder should be sent.

**Table 4:** Evaluation of the 3-day appointment reminder, among parents who reported receiving the 3-day reminder (n=176) and public health center staff who completed the survey (n=22).

Variable				Response, n (%)				
				Mill Woods		Lethbridge		Total
**Parents**								
	**3-day reminder was helpful to remember the appointment date and time (n=176)**							
		Yes	89 (98.9)		86 (100)		175 (99.4)	
		No	1 (1.1)		0 (0)		1 (0.6)	
	**Impact of the 3-day reminder (n=176)**							
		Changed appointment	7 (7.8)		5 (5.8)		12 (6.8)	
		Did nothing	82 (91.1)		79 (91.9)		161 (91.4)	
		Other	1 (1.1)		2 (2.3)		3 (1.7)	
	**Best time to send the reminder (n=222)^a^**							
		On the same day as the appointment	9 (8.6)		9 (7.7)		18 (8.1)	
		1 day before the appointment	23 (21.9)		30 (25.6)		53 (23.9)	
		2 days before the appointment	16 (15.2)		23 (19.7)		39 (17.6)	
		3 days before the appointment	53 (50.5)		60 (51.3)		113 (50.9)	
		1 week before the appointment	36 (34.3)		49 (41.9)		85 (38.3)	
		2 weeks before the appointment	8 (7.6)		3 (2.6)		11 (5.0)	
		1 month before the appointment	10 (9.5)		7 (6.0)		17 (7.7)	
		Other	0 (0)		2 (1.7)		2 (0.9)	
	**Attendance at the 18-month immunization appointment (n=222)^b^**							
		Yes	99 (94.3)		109 (93.2)		208 (93.6)	
		No	6 (5.7)		8 (6.8)		14 (6.3)	
**Public health center staff (n=22)**								
	**3-day reminder was helpful**							
		Yes	11 (91.7)		10 (100)		21 (95.5)	
		No	0 (0)		0 (0)		0 (0)	
		Not sure or I do not know	1 (8.3)		0 (0)		1 (4.5)	
	**Best time to send reminder**							
		1 week before the visit	0 (0)		2 (20)		2 (9.1)	
		3 days before the visit	7 (58.3)		5 (50)		12 (54.6)	
		2 days before the visit	2 (16.7)		1 (10)		3 (13.6)	
		1 day before the visit	3 (25)		0 (0)		3 (13.6)	
		Other	0 (0)		1 (10)		1 (4.6)	
	**How many date and time appointments should be sent**							
		1	6 (50)		7 (70)		13 (59.1)	
		2	4 (33.3)		2 (20)		6 (27.3)	
		3	2 (16.7)		1 (10)		3 (13.6)	

^a^Answered by all parents, not just those who received the 3-day reminder.

^b^One respondent did not specify their other response, and the other respondent specified that no reminders should be sent.

### Web-Based Immunization Information

#### Survey Data

Approximately half (51/114, 44.7%) of the parents who received the 15-month reminder reported reading the web-based immunization information (Table 5). Most participants read this information in English (47/51, 92%), found it helpful to read in their language of choice (51/51, 100%), and felt more prepared for their child’s appointment (50/51, 98%). The most common reason for not reading the information was already knowing the information (28/63, 44%). For parents who did not receive the 15-month reminder (108/222, 48.6%; data not shown), most reported that reading immunization information in their language of choice would be helpful (97/108, 89.8%) and that they would be more prepared for their child’s appointment (81/108, 75%).

As shown in Table 5, some PHC staff reported that more clients read the immunization information sheets than usual (6/19, 32%), more clients engaged in conversation about vaccines (4/19, 21%), and more clients asked questions about vaccines (6/19, 31%). Others reported that they noticed no changes in clients reading the immunization information sheets (7/19, 37%), conversations about vaccines during the visit (8/19, 42%), or the efficiency of exchanging knowledge with clients during the visit (6/19, 31%).

**Table 5:** Evaluation of immunization information sheet use, among parents who reported receiving the 15-month reminder (n=114) and public health center staff (n=19).

Variable				Response, n (%)				
				Mill Woods		Lethbridge		Total
**Parents**								
	**Read web-based information about vaccines (n=114)**							
		Yes	31 (52.5)		20 (36.4)		51 (44.7)	
		No	28 (47.5)		35 (63.6)		63 (55.3)	
	**Language or languages read (n=51)^a,b,c^**							
		English	29 (93.5)		18 (90)		47 (92.2)	
		Punjabi	6 (19.4)		1 (5)		7 (13.7)	
		Tagalog	2 (6.5)		0 (0)		2 (3.9)	
		Spanish	0 (0)		2 (10)		2 (3.9)	
		French	1 (3.2)		0 (0)		1 (2)	
	**Helpful to read immunization information in language of choice (n=51)^a^**							
		Yes	31 (100)		20 (100)		51 (100)	
		No	0 (0)		0 (0)		0 (0)	
	**After reading the immunization information, felt more prepared for child’s appointment (n=51)^a^**							
		Yes	30 (96.8)		20 (100)		50 (98)	
		No	1 (3.2)		0 (0)		1 (2)	
	**Reasons for not reading the web-based immunization information (n=63)^b,d^**							
		Already knew the information	11 (39.3)		17 (48.6)		28 (44.4)	
		Did not see a link to information in reminder	5 (17.9)		1 (2.9)		6 (9.5)	
		Too long	2 (7.1)		2 (5.7)		4 (6.3)	
		Forgot	2 (7.1)		1 (2.9)		3 (4.8)	
		Not enough time	0 (0)		3 (8.6)		3 (4.8)	
		Language of choice not available	1 (3.6)		1 (2.9)		2 (3.2)	
		Felt information was unnecessary, as had already decided to get child immunized	2 (7.1)		0 (0)		2 (3.2)	
		Felt doctors or nurses would provide the information	2 (7.1)		0 (0)		2 (3.2)	
		Had no concerns with immunization	0 (0)		2 (5.7)		2 (3.2)	
		Difficult to understand	0 (0)		1 (2.9)		1 (1.6)	
		Font too small on device screen	0 (0)		1 (2.9)		1 (1.6)	
		Did not specify	3 (10.7)		8 (22.9)		11 (17.5)	
**PHC^e^** **staff: nurses**								
	**Impact of offering immunization information sheets in other languages (n=19)**							
		I did not notice any change	6 (50)		1 (10)		7 (36.8)	
		More clients read the information sheets than usual	4 (33.3)		2 (20)		6 (31.6)	
		Not sure or I do not know	2 (16.7)		2 (20)		4 (21.1)	
		Other	0 (0)		2 (20)		2 (10.5)	
	**Impact of offering immunization information sheets in other languages on the conversation about vaccines during the visit (n=19)**							
		I did not notice any change	6 (50)		2 (20)		8 (42.1)	
		I noticed more clients engaging in the conversation than usual	4 (33.3)		0 (0)		4 (21.1)	
		Not sure or I do not know	2 (16.7)		5 (50)		7 (36.8)	
	**Impact of offering immunization information sheets in other languages on the efficiency of exchanging knowledge with clients during the visit (n=19)**							
		I do not think it changed anything	5 (41.7)		1 (10)		6 (31.2)	
		I noticed more clients asking questions about vaccines than usual	5 (41.7)		1 (10)		6 (31.2)	
		Not sure or I do not know	2 (16.7)		5 (50)		7 (36.8)	
	**How to increase use of the immunization information sheets (n=22)^b,f^**							
		Provide the link to the website in the appointment reminder text (ie, the 3-day reminder)	9 (75)		8 (80)		17 (77.3)	
		Promote the website using posters or handouts in the health center	9 (75)		7 (70)		16 (72.7)	
		Have printed copies of the information sheets (in various languages) available at the health center	6 (50)		6 (60)		12 (54.5)	
		Other	0 (0)		1 (10)		1 (4.5)	

^a^For parents who reported reading web-based immunization information (Mill Woods: n=31, Lethbridge: n=20).

^b^Respondents could select more than one option.

^c^Other potential language options included Arabic, Chinese, Italian, and Vietnamese; however, these options were not selected by any parent.

^d^For parents who reported that they did not read web-based immunization information (Mill Woods: n=28, Lethbridge: n=35).

^e^PHC: public health center.

^f^Question asked to all public health center staff (n=22).

#### Web-Based Access Data

According to Google Analytics, ChIRP web-based immunization information pages received 207 unique visits during the intervention period. Immunization information was most often read in English (118/207, 57%), followed by Punjabi (52/207, 25.1%), Arabic (13/207, 6.3%), Spanish (12/207, 5.8%), Italian (4/207, 1.9%), Chinese (4/207, 1.9%), French (2/207, 1%), Tagalog (1/207, 0.5%), and Vietnamese (1/207, 0.5%).

## Discussion

This pilot intervention aimed to assess the effectiveness and acceptability of SMS text message reminders for preschool immunization appointments. Consistent with previous literature [19,27], our study suggests that SMS text message reminders can reduce appointment no-shows and are acceptable to parents and health service providers.

### No-show Rates

There was a decline in absolute no-show rates at Mill Woods PHC, which corresponds with other studies [28,29] reporting that SMS text message reminders improved immunization appointment attendance. Lethbridge PHC did not exhibit a decline in no-shows; however, they had higher attendance before the intervention, likely because of preexisting strategies at that site (eg, manual reminders) as indicated by K Jong (personal communication, June 15, 2020), so perhaps the intervention had less impact.

### SMS Text Message Reminders

Overall, parents reported high acceptability of the 15-month and 3-day message reminders, with almost all stating that they were helpful. Literature has shown that parents often prefer SMS text message reminders over mail or email because of the convenience and timeliness [27,30,31]. Jacobson Vann et al [3] found that SMS text message reminders increased the booking of immunization visits as they acted as a call to action for parents. In our study, a third of the surveyed parents reported booking or rescheduling their child’s 18-month immunization appointment after receiving the 15-month reminder. It is possible that these parents may have missed the appointments had they not received a reminder [32]. However, most surveyed parents reported no action upon receiving the 15-month or 3-day reminders, which corresponds with the small changes in no-shows observed in this study. Overall, most parents reported that their child received their 18-month immunization as scheduled. Improvement in the timely receipt of childhood vaccines minimizes risks for vaccine-preventable diseases [30] and may reduce extra work to recall parents [33].

Most of the surveyed PHC staff stated that SMS text message reminders were helpful, indicating provider support for the intervention. This reflects the readiness to engage parents in positive discussions about childhood immunizations and encourage them to subscribe to reminder services [34]. In addition, most PHC staff reported that more clients came to their scheduled immunizations than usual during the intervention. Our no-show analysis revealed a significant improvement in appointment attendance at Mill Woods PHC following the intervention. PHC staff from both sites supported the expansion of this intervention to the 2-month and 12-month immunization programs.

Parents and PHC staff agreed that the best time to send appointment reminders was 3 days before the appointment but had different preferences for the booking (15-month) reminder, with staff preferring the reminder to be sent earlier. This difference in preferences is likely because of staff needing to schedule in advance to accommodate many immunization appointments, whereas parents may not be thinking about the 18-month appointment until their child is 17 months old or may forget the appointment if the reminder is sent too early. Interestingly, the staff at Mill Woods PHC preferred the reminder to be sent earlier than the Lethbridge staff. This may reflect differences in the size of the 2 PHCs; Mill Woods serves a larger urban area and thus requires parents to book in advance, whereas Lethbridge serves a smaller urban population within a rural zone and may accommodate appointments on shorter notice.

### Web-Based Immunization Information

The web-based immunization information in different languages was also positively received by parents. According to the Google Analytics data, many participants accessed the information in other languages. There is increasing awareness that language barriers impede immunization service delivery, but they continue to remain unaddressed in many existing reminder and recall systems [33]. Our study and previous work [35] have shown that parents favor language-specific immunization information.

Notably, the Google Analytics data showed a different picture of website activity than the parent survey. Specifically, Google Analytics showed more visits to the website (n=207) compared with the number of survey participants who reported accessing the information (n=51). As the link to the website was sent to all parents receiving the intervention, it is possible that nonsurvey participants accessed the information. In addition, Google Analytics showed more diversity in the languages accessed on the website (ie, more non-English users) compared with the parent survey. This may reflect the fact that English-speaking participants may have been more likely to complete the survey than participants whose first language was not English. The diverse languages accessed by parents suggest the need to provide appropriately translated immunization information.

### Implications

Using SMS text message reminders for immunization appointments may be a convenient and cost-effective way of reducing appointment no-shows. The acceptability of the intervention by parents and PHC staff means that there is potential for SMS text message reminders to be implemented for other immunization programs, particularly 2-month and 12-month immunizations, as well as in other provinces. Future research should consider the use of experimental studies to evaluate the impact of SMS text message reminders on immunization coverage following widespread implementation.

To maximize the effectiveness of an SMS text message reminder system, it is important to make it appealing to both parents and PHC staff. For example, parents preferred later booking reminders than staff; therefore, perhaps sending both early and later reminders might be a useful compromise.

### Strengths and Limitations

A strength of this study is the diverse perspectives obtained from both parents and PHC staff at 2 different sites in Alberta: one large urban site and one small urban site in a rural area. We were also able to assess the change in no-show rates at both clinics, using data over an extended time (ie, 1 year for both baseline and intervention), and in comparison with a control site for each. The limitations of this study include a low parent survey response rate (222/929, 23.9%), which may be because of the 7-month gap between recruitment and when links to the evaluation survey were sent out. It is possible that parents who responded to the survey differed from nonrespondents. For example, as the survey was conducted in English, non-English speakers were likely to be underrepresented. In addition, a lower proportion of survey respondents did not attend their child’s 18-month immunization appointment compared with the calculated no-show rates at the 2 clinics, which means that our survey likely underrepresented no-shows. However, it is encouraging that the survey respondents had diverse sociodemographic characteristics, such as income. The number of surveyed PHC staff was also low, which may be explained by the increased strain on health care providers during the COVID-19 pandemic [36]. In addition, at the onset of the pandemic, reminders were paused for 1 month, while PHCs adapted to new ways of service delivery. In addition, we cannot definitively attribute the decline in no-show rates to the intervention. However, our comparison over time and between PHCs gives us some confidence. As more parents reported receiving the 3-day reminder compared with the 15-month reminder, it is possible that parents may have forgotten about the 15-month reminder, which was sent 4 months before the evaluation, compared with the 3-day reminder, sent 1 month prior. Finally, as the intervention and evaluation were only carried out at 2 PHCs in Alberta, the generalizability may be limited. However, this was a pilot study to determine acceptability, with the potential to carry out large-scale interventions and evaluations in the future.

### Conclusions

This study found that parents and staff at the 2 PHCs were highly accepting of the SMS text message reminder system implemented to address the drop in coverage for 18-month immunizations. The intervention reduced the number of missed appointments at the urban intervention site. Findings support the use of SMS text message reminders as a convenient and acceptable method to minimize parental forgetfulness and potentially reduce appointment no-shows.

## Abbreviations

AHS: Alberta Health Services
ChIRP: Childhood Immunization Reminder Project
DTaP-IPV-Hib: diphtheria-tetanus-acellular pertussis-polio-Haemophilus influenzae type b
MMRV: measles-mumps-rubella-varicella
PHC: public health center
REDCap: Research Electronic Data Capture

## References

[1] (2019). Immunization. World Health Organization.

[2] (2020). Vaccine Coverage in Canadian Children: Results from the 2017 Childhood National Immunization Coverage Survey (cNICS). Government of Canada.

[3] Jacobson Vann JC, Jacobson RM, Coyne-Beasley T, Asafu-Adjei JK, Szilagyi PG (2018). Patient reminder and recall interventions to improve immunization rates. Cochrane Database Syst Rev.

[4] Kershaw T, Suttorp V, Simmonds K, St Jean T (2014). Outbreak of measles in a non-immunizing population, Alberta 2013. Can Commun Dis Rep.

[5] (2020). Highlights from the 2019 childhood National Immunization Coverage Survey (cNICS). Government of Canada.

[6] Hill HA, Yankey D, Elam-Evans LD, Singleton JA, Sterrett N (2021). Vaccination coverage by age 24 months among children born in 2017 and 2018 - National Immunization Survey-Child, United States, 2018-2020. MMWR Morb Mortal Wkly Rep.

[7] (2021). Immunization: percent of children vaccinated by age 24 months. National Center for Health Statistics - Centers for Disease Control and Prevention.

[8] (2021). Recommended immunization schedules: Canadian Immunization Guide. Government of Canada.

[9] (2021). Interactive Health Data Application. Government of Alberta.

[10] Bauer KE, Agruss JC, Mayefsky JH (2020). Partnering with parents to remove barriers and improve influenza immunization rates for young children. J Am Assoc Nurse Pract.

[11] Wójcik OP, Simonsen J, Mølbak K, Valentiner-Branth P (2013). Validation of the 5-year tetanus, diphtheria, pertussis and polio booster vaccination in the Danish childhood vaccination database. Vaccine.

[12] Bell CA, Simmonds KA, MacDonald SE (2015). Exploring the heterogeneity among partially vaccinated children in a population-based cohort. Vaccine.

[13] Robinson JL (2018). Potential strategies to improve childhood immunization rates in Canada. Paediatr Child Health.

[14] Guttmann A, Manuel D, Dick P, To T, Lam K, Stukel TA (2006). Volume matters: physician practice characteristics and immunization coverage among young children insured through a universal health plan. Pediatrics.

[15] Kempe A, Stockwell MS, Szilagyi P (2021). The contribution of reminder-recall to vaccine delivery efforts: a narrative review. Acad Pediatr.

[16] Hofstetter AM, DuRivage N, Vargas CY, Camargo S, Vawdrey DK, Fisher A, Stockwell MS (2015). Text message reminders for timely routine MMR vaccination: a randomized controlled trial. Vaccine.

[17] Kharbanda EO, Stockwell MS, Fox HW, Andres R, Lara M, Rickert VI (2011). Text message reminders to promote human papillomavirus vaccination. Vaccine.

[18] (2021). Communicating effectively about immunization: Canadian Immunization Guide. Government of Canada.

[19] Hall AK, Cole-Lewis H, Bernhardt JM (2015). Mobile text messaging for health: a systematic review of reviews. Annu Rev Public Health.

[20] Wynn CS, Catallozzi M, Kolff CA, Holleran S, Meyer D, Ramakrishnan R, Stockwell MS (2021). Personalized reminders for immunization using short messaging systems to improve human papillomavirus vaccination series completion: parallel-group randomized trial. JMIR Mhealth Uhealth.

[21] Rod MH, Ingholt L, Bang Sørensen B, Tjørnhøj-Thomsen T (2014). The spirit of the intervention: reflections on social effectiveness in public health intervention research. Crit Public Health.

[22] (2022). Alberta Regional Dashboard. Government of Alberta.

[23] R Core Team (2020). R: a language and environment for statistical computing. R Foundation for Statistical Computing.

[24] Harris PA, Taylor R, Thielke R, Payne J, Gonzalez N, Conde JG (2009). Research electronic data capture (REDCap)--a metadata-driven methodology and workflow process for providing translational research informatics support. J Biomed Inform.

[25] Google Analytics. Google.

[26] (2019). IBM SPSS Statistics for Windows, Version 26.0. IBM Corp.

[27] Domek GJ, Contreras-Roldan IL, O'Leary ST, Bull S, Furniss A, Kempe A, Asturias EJ (2016). SMS text message reminders to improve infant vaccination coverage in Guatemala: a pilot randomized controlled trial. Vaccine.

[28] Lin CL, Mistry N, Boneh J, Li H, Lazebnik R (2016). Text message reminders increase appointment adherence in a pediatric clinic: a randomized controlled trial. Int J Pediatr.

[29] Oladepo O, Dipeolu IO, Oladunni O (2021). Outcome of reminder text messages intervention on completion of routine immunization in rural areas, Nigeria. Health Promot Int.

[30] Ahlers-Schmidt CR, Chesser AK, Nguyen T, Brannon J, Hart TA, Williams KS, Wittler RR (2012). Feasibility of a randomized controlled trial to evaluate Text Reminders for Immunization Compliance in Kids (TRICKs). Vaccine.

[31] Kharbanda EO, Stockwell MS, Fox HW, Rickert VI (2009). Text4Health: a qualitative evaluation of parental readiness for text message immunization reminders. Am J Public Health.

[32] McLean SM, Booth A, Gee M, Salway S, Cobb M, Bhanbhro S, Nancarrow SA (2016). Appointment reminder systems are effective but not optimal: results of a systematic review and evidence synthesis employing realist principles. Patient Prefer Adherence.

[33] Jong KM, Sikora CA, MacDonald SE (2021). Childhood immunization appointment reminders and recalls: strengths, weaknesses and opportunities to increase vaccine coverage. Public Health.

[34] Hofstetter AM, Vargas CY, Kennedy A, Kitayama K, Stockwell MS (2013). Parental and provider preferences and concerns regarding text message reminder/recall for early childhood vaccinations. Prev Med.

[35] de Nuncio ML, Price SA, Tjoa T, Lashuay N, Jones MC, Elder JP (1999). Pretesting Spanish-language educational radio messages to promote timely and complete infant immunization in California. J Community Health.

[36] Barrett K, Khan YA, Mac S, Ximenes R, Naimark DM, Sander B (2020). Estimation of COVID-19-induced depletion of hospital resources in Ontario, Canada. CMAJ.

